# Supplementary material for: Systems-level exploitation of OxyR regulon unravels a potential antibacterial target in Pseudomonas aeruginosa
Source: Commun Biol. 2025 Sep 26;8:1370. doi: 10.1038/s42003-025-08770-w (PMC12475218; doi:10.1038/s42003-025-08770-w)
Supplement: Supplementary file 1 — Supplementary Information [file 42003_2025_8770_MOESM1_ESM.pdf]

Supplementary information

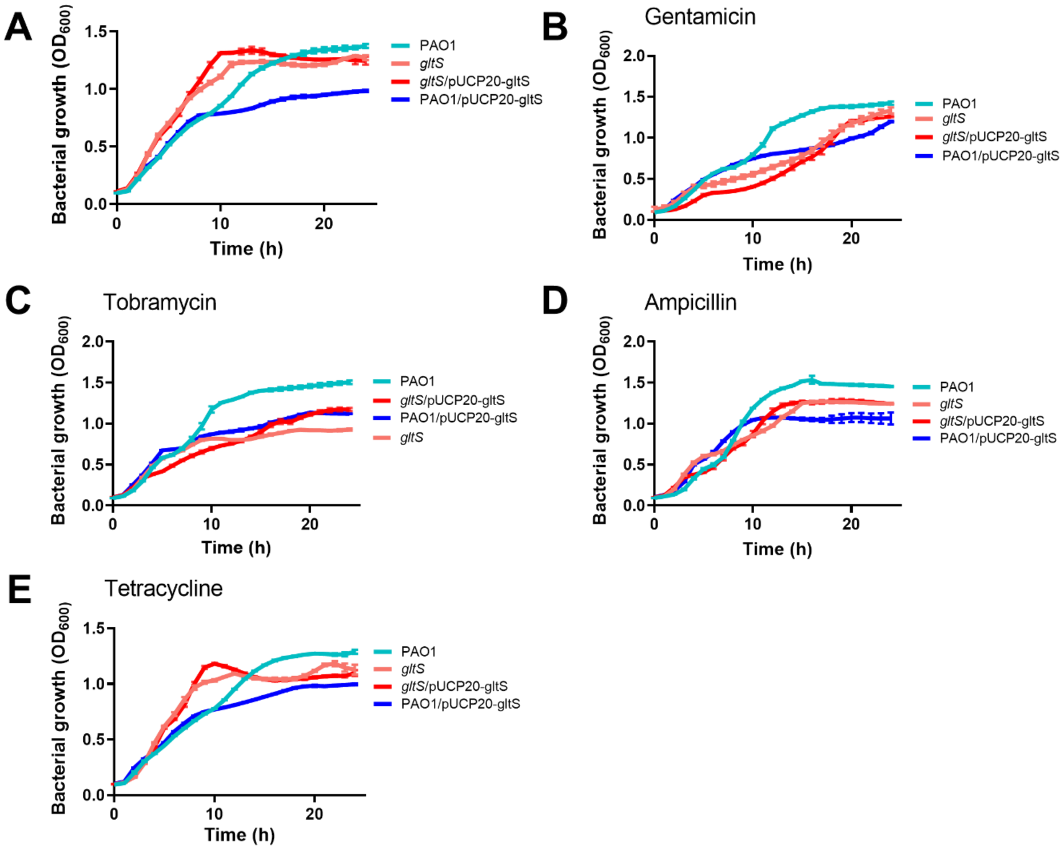

Supplementary Figure 1. Growth curve analysis of *gltS*, PAO1 and its derivatives. (A) Growth curve analysis of PAO1, *gltS*, *gltS*/pUCP20-gltS and PAO1/pUCP20-gltS, under normal LB liquid medium growth condition. Growth curve of PAO1, *gltS*, *gltS*/pUCP20-gltS and PAO1/pUCP20-gltS was also detected under 2 μg/ml of gentamicin (B), 0.2 μg/ml of tobramycin (C), 128 μg/ml of ampicillin (D) and 4 μg/ml of tetracycline (E) to observe the difference of fitness for PAO1 derivatives.

Supplementary Table 2. Selected genes involved in energy metabolism and putative enzymes.

PA no.	Gene	FC	*P* value	QS	Product description
Energy metabolism					
PA4133		-4.87	0.000	+	Cytochrome c oxidase subunit (cbb3-type)
PA0113		-2.83	0.000		Probable cytochrome c oxidase assembly factor
PA3032	*snr1*	-2.78	0.000	+	Cytochrome c Snr1
PA0525		-2.41	0.000		Probable dinitrification protein NorD
PA0106	*coxA*	-2.30	0.000	+	Cytochrome c oxidase, subunit I
PA3415		-2.20	0.001		Probable dihydrolipoamide acetyltransferase
PA1319	*cyoC*	-2.18	0.007	+	Cytochrome o ubiquinol oxidase subunit III
PA1318	*cyoB*	-1.88	0.000	+	Cytochrome o ubiquinol oxidase subunit I
PA0523	*norC*	-1.86	0.000		Nitric-oxide reductase subunit C
PA3417		-1.84	0.004		Probable pyruvate dehydrogenase E1 component, alpha subunit
PA0107		-1.83	0.002	+	Conserved hypothetical protein
PA0524	*norB*	-1.73	0.000		Nitric-oxide reductase subunit B
PA1317	*cyoA*	-1.71	0.000	+	Cytochrome o ubiquinol oxidase subunit II
PA0105	*coxB*	-1.70	0.000	+	Cytochrome c oxidase, subunit II
PA1174	*napA*	-1.68	0.000		Periplasmic nitrate reductase protein NapA
PA0108	*coIII*	-1.67	0.000	+	Cytochrome c oxidase, subunit III
PA1172	*napC*	-1.54	0.000		Cytochrome c-type protein NapC
PA5300	*cycB*	-1.31	0.000		Cytochrome c5
PA3392	*nosZ*	-1.28	0.000	+	Nitrous-oxide reductase precursor
PA1173	*napB*	-1.26	0.002	+	Cytochrome c-type protein NapB precursor
PA3930	*cioA*	-1.15	0.000		Cyanide insensitive terminal oxidase
PA0195	*pntAA*	-1.06	0.000		Putative NAD(P) transhydrogenase, subunit alpha part 1
PA1562	*acnA*	-1.02	0.000		Aconitate hydratase 1
PA3878	*narX*	1.21	0.000		Two-component sensor NarX
Putative enzymes					
PA2069		-7.57	0.000	+	Probable carbamoyl transferase
PA2302	*ambE*	-6.74	0.000	+	Probable non-ribosomal peptide synthetase
PA2305	*ambB*	-6.54	0.000	+	Probable non-ribosomal peptide synthetase
PA3330		-6.39	0.000	+	Probable short chain dehydrogenase
PA4217	*phzS*	-6.30	0.000	+	Flavin-containing monooxygenase
PA3535		-6.09	0.000	+	Probable serine protease
PA3328		-5.92	0.000	+	Probable FAD-dependent monooxygenase
PA2587	*pqsH*	-5.52	0.000	+	Probable FAD-dependent monooxygenase
PA1914		-4.63	0.000	+	Conserved hypothetical protein
PA4209	*phzM*	-4.53	0.000	+	Probable phenazine-specific methyltransferase
PA4131		-3.88	0.000	+	Probable iron-sulfur protein
PA1893		-3.35	0.000	+	Hypothetical protein
PA1662	*clpV2*	-3.07	0.000	+	Probable ClpA/B-type protease
PA5181		-2.99	0.000		Probable oxidoreductase

PA4041		-2.71	0.002		Hypothetical protein
PA3957		-2.53	0.005		Probable short-chain dehydrogenase
PA4190	*pqsL*	-2.36	0.000	+	Probable FAD-dependent monooxygenase
PA2326		-2.20	0.007		Hypothetical protein
PA1881		-1.90	0.003	+	Probable oxidoreductase
PA2024		-1.87	0.007		Probable ring-cleaving dioxygenase
PA1860		-1.86	0.001		Hypothetical protein
PA1137		-1.86	0.000		Probable oxidoreductase
PA3723		-1.77	0.000		Probable FMN oxidoreductase
PA0704		-1.64	0.000		Probable amidase
PA1169		-1.55	0.003		Probable lipoxygenase
PA0333		-1.50	0.003		Hypothetical protein
PA1880		-1.36	0.000		Probable oxidoreductase
PA1885		-1.32	0.006		Conserved hypothetical protein
PA3426		-1.30	0.006		Probable enoyl CoA-hydratase/isomerase
PA4202	*nmoA*	-1.24	0.000		Hypothetical protein
PA4786		-1.17	0.000		Probable short-chain dehydrogenase
PA0364		-1.07	0.000	+	Probable oxidoreductase
PA5546		-1.03	0.000		Conserved hypothetical protein

Supplementary Table 3. Selected genes involved in transport of small molecules.

PA no.	Gene	FC	*P* value	QS	Product description
PA3176	*gltS*	3.35	0.001		Glutamate/sodium ion symporter, GltS
PA2338		2.90	0.000	+	Probable binding protein component of ABC maltose/mannitol transporter
PA2341		2.88	0.000	+	Probable ATP-binding component of ABC maltose/mannitol transporter
PA2398	*fpvA*	1.98	0.000		Ferripyoverdine receptor
PA1260	*lhpP*	1.75	0.000		Amino acid ABC transporter periplasmic binding protein
PA1074	*braC*	1.42	0.001		Branched-chain amino acid transport protein BraC
PA5082	*dguC*	1.37	0.000		Probable binding protein component of ABC transporter
PA2558		1.35	0.006		Probable transport protein
PA2760	*oprQ*	1.33	0.000		Probable outer membrane protein precursor
PA4501	*opdD*	1.23	0.000		Glycine-glutamate dipeptide porin OpdP
PA5153		1.20	0.002		Amino acid (lysine/arginine/ornithine/histidine/octopine) ABC transporter periplasmic binding protein
PA0783	*putP*	1.18	0.000		Sodium/proline symporter PutP
PA3766		1.18	0.001		Probable aromatic amino acid transporter
PA1651		1.16	0.002		Probable transporter
PA4023		1.14	0.001		Probable transport protein
PA5169	*dctM*	1.11	0.003		Probable C4-dicarboxylate transporter
PA4514		1.11	0.000		Probable outer membrane receptor for iron transport
PA3038	*opdQ*	1.10	0.000	+	Probable porin
PA1288		1.09	0.000		Probable outer membrane protein precursor
PA5167	*dctP*	1.08	0.000		Probable C4-dicarboxylate-binding protein
PA4675	*chtA*	1.08	0.000		Probable TonB-dependent receptor
PA3234		1.07	0.000	+	Probable sodium:solute symporter
PA5530		1.05	0.001		Probable MFS dicarboxylate transporter
PA2533		1.04	0.000		Probable sodium:alanine symporter
PA4358	*feoB*	1.01	0.000		Probable ferrous iron transport protein
PA2711		1.01	0.003	+	Probable periplasmic spermidine/putrescine-binding protein
PA1339	*aatP*	-1.01	0.000		Amino acid ABC transporter ATP binding protein
PA4589		-1.17	0.001		Probable outer membrane protein precursor
PA3531	*bfrB*	-1.23	0.000		Bacterioferritin
PA2435		-1.26	0.001		Probable cation-transporting P-type ATPase
PA2291		-1.33	0.000		Probable glucose-sensitive porin
PA5158		-1.63	0.002		Probable outer membrane protein precursor
PA1041		-1.79	0.000		Probable outer membrane protein precursor
PA4207	*mexI*	-1.89	0.000	+	Probable Resistance-Nodulation-Cell Division (RND) efflux transporter
PA3692	*lptF*	-2.08	0.000	+	Lipotoxon F, LptF
PA3522	*mexQ*	-2.12	0.002		Probable Resistance-Nodulation-Cell Division (RND) efflux transporter

PA4080		-2.53	0.001		Probable response regulator
PA4590	*pra*	-2.75	0.000	+	Protein activator
PA4206	*mexH*	-2.84	0.000	+	Probable Resistance-Nodulation-Cell Division (RND) efflux membrane fusion protein precursor
PA3920		-2.93	0.000		Probable metal transporting P-type ATPase
PA0103		-3.00	0.002		Probable sulfate transporter
PA2592		-3.09	0.000	+	Probable periplasmic spermidine/putrescine-binding protein
PA3523	*mexP*	-3.35	0.002		Probable Resistance-Nodulation-Cell Division (RND) efflux membrane fusion protein precursor
PA1212		-3.93	0.003	+	Probable major facilitator superfamily (MFS) transporter
PA3336		-5.41	0.000	+	Probable major facilitator superfamily (MFS) transporter

Supplementary Table 4. Full list of differentially expressed metabolites in the *oxyR* as compared to its wild-type PAO1.

id	MS2 name	MS2 score	*P* VALUE	FC	LogFC
11875	1-Palmitoyl-2-hydroxy-sn-glycero-3-phosphoethanolamine	0.9921	0.037	0.509	-0.975
24	2-Amino-2-methyl-1,3-propanediol	0.8336	0.007	1.436	0.522
5508	2-C-Methyl-D-erythritol 2,4-cyclodiphosphate	0.8487	0.016	1.322	0.403
9251	2'-Deoxyguanosine 5'-monophosphate (dGMP)	0.9772	0.001	1.363	0.446
1207	2-Phenylacetamide	0.9898	0.014	1.099	0.136
2046	3-(3-Indolyl)-2-oxopropanoic acid	0.8315	0.032	1.345	0.428
2510	3,4-Dihydroxy-L-phenylalanine(L-DOPA)	0.7905	0.039	1.549	0.631
2764	3-Hydroxymandelic acid	0.9995	0.026	1.562	0.643
7165	3'-O-methyladenosine	0.9993	0.018	1.491	0.577
2498	5-L-Glutamyl-L-alanine	0.959	0.032	2.587	1.371
7208	5'-Phosphoribosyl-5-amino-4-imidazolecarboxamide (AICAR)	0.8407	0.008	1.255	0.328
9718	Adenosine 3'-monophosphate	0.9995	0.002	1.371	0.455
13671	Adenosine 5'-triphosphate (ATP)	0.7531	0.025	0.753	-0.409
8369	Adenosine monophosphate (AMP)	0.9799	0.002	1.384	0.469
8145	Adenosine monophosphate (AMP)	0.9999	0.005	1.383	0.468
6990	Ajmalicine	0.9861	0.000	3.429	1.778
6208	alpha-Linolenic acid	0.8873	0.040	0.854	-0.228
5900	Capsaicin	0.7999	0.027	1.417	0.502
19079	Coproporphyrin III	0.8725	0.004	1.272	0.348
7302	Cytidine 5'-monophosphate (CMP)	0.9102	0.000	1.364	0.448
1425	D-(+)-Galactosamine	0.85	0.007	1.400	0.485
777	D-Aspartic acid	0.9929	0.033	1.302	0.380
25628	Decanoyl-CoA	0.9989	0.001	0.548	-0.869
25524	Decanoyl-CoA	0.9229	0.000	0.536	-0.900
3380	Deoxycytidine	0.9967	0.012	1.380	0.465
14396	Deoxyguanosine triphosphate (dGTP)	0.9541	0.008	0.706	-0.502
7272	Deoxythymidine 5'-phosphate (dTMP)	0.9329	0.003	1.370	0.454
7197	Deoxythymidine 5'-phosphate (dTMP)	0.9986	0.006	1.366	0.450
14835	D-Glucose 6-phosphate	0.7462	0.002	1.674	0.744
1077	Diacetyl	0.7207	0.039	1.506	0.591
1605	Diphenylamine	0.8698	0.013	0.736	-0.442
1519	DL-2-Aminoadipic acid	0.9363	0.014	1.266	0.340
2684	DL-Phenylalanine	0.9789	0.001	1.870	0.903
7291	D-Mannitol 1-phosphate	0.7003	0.013	2.900	1.536
2594	Dodecanoic acid	0.707	0.001	1.423	0.509
211	D-Ornithine	0.9998	0.005	1.392	0.477
1344	Fosfomycin	0.9217	0.009	1.334	0.415
5520	gamma-Glutamyl-L-methionine	0.9173	0.000	1.256	0.329
5412	gamma-L-Glutamyl-L-glutamic acid	0.9866	0.001	1.319	0.399
5437	gamma-L-Glutamyl-L-glutamic acid	0.9242	0.001	1.291	0.369
65	Glycine	0.9988	0.001	1.376	0.460
4349	Guanidineacetic acid	0.6646	0.036	1.123	0.168
1951	Harmane	0.9793	0.000	1.592	0.671
2881	Harmine	0.9095	0.000	1.670	0.739
7712	His-Asp	0.9941	0.024	1.914	0.937

5210	Ile-Pro	0.8115	0.022	1.337	0.419
1887	Indoleacetic acid	0.766	0.034	1.497	0.582
5353	Kinetin	0.9914	0.000	1.813	0.858
3511	Kynurenic acid	0.9923	0.040	1.327	0.409
6585	L-Arabitol	0.739	0.035	1.530	0.613
824	L-Aspartate	0.9849	0.029	1.280	0.356
5589	L-Aspartyl-L-phenylalanine	0.9786	0.047	2.051	1.036
1186	L-Glutamate	0.9962	0.029	1.120	0.164
1079	L-Glutamine	0.9895	0.004	1.726	0.788
1266	L-Histidine	0.8096	0.025	1.198	0.261
2084	L-Iditol	0.8336	0.002	1.712	0.776
7891	Linoleoyl ethanolamide	0.6979	0.027	0.645	-0.634
735	L-Pipecolic acid	0.9997	0.032	1.495	0.580
726	L-Pyroglutamic acid	0.9789	0.009	1.141	0.190
6135	L-Threonate	0.6693	0.009	1.755	0.812
23508	Maltopentaose	0.7736	0.048	1.844	0.883
3482	Myristic acid	0.983	0.008	1.229	0.297
3393	Myristoleic acid	0.9972	0.001	1.803	0.850
2880	N-(omega)-Hydroxyarginine	0.9853	0.016	1.920	0.941
6691	N-Acetylneuraminic acid	0.6634	0.041	1.813	0.858
3096	N-Acetylserotonin	0.915	0.003	0.571	-0.809
10480	Nafcillin	0.9128	0.043	1.901	0.927
621	Nicotinate	0.9986	0.010	1.569	0.650
1578	Norharmane	0.9897	0.006	1.257	0.329
6148	O-Acetyl-L-serine	0.7251	0.021	1.784	0.835
24864	Octanoyl-CoA	0.9279	0.016	0.593	-0.753
17826	Oxycodone	0.98	0.005	6.831	2.772
5525	Pantetheine	0.9152	0.032	2.254	1.173
4721	Pectin (Galacturonic acid)	1	0.021	1.345	0.428
3992	Pentadecanoic Acid	0.976	0.009	1.446	0.532
8508	PGE3	0.7876	0.004	2.354	1.235
6427	Phenethyl Caffeiate	0.9993	0.039	2.018	1.013
6081	Phe-Tyr	0.8736	0.003	1.370	0.455
6957	Picrotoxinin	0.9953	0.010	1.175	0.232
4637	Prilocaine	0.7139	0.001	2.005	1.003
2924	Pro-Gly	0.9923	0.035	1.483	0.569
9688	Salicyluric acid	0.995	0.023	0.653	-0.615
8022	sn-Glycerol 1-phosphate	0.981	0.009	2.138	1.096
4851	Stavudine	0.9907	0.004	1.253	0.326
13202	Stearoylcarnitine	0.9888	0.048	0.560	-0.836
5661	Temazepam	0.6303	0.007	1.248	0.320
8835	Trp-Tyr	0.8129	0.004	1.244	0.314
6157	Tyr-Leu	0.9825	0.029	1.293	0.371
390	Uracil	0.9669	0.047	1.303	0.382
16845	Uridine 5'-diphosphoglucuronic acid (UDP-D-glucuronate)	0.782	0.015	1.186	0.246
7244	Uridine 5'-monophosphate (UMP)	0.9958	0.000	1.410	0.496
3564	Val-Asn	0.8822	0.041	2.260	1.176
4864	Val-Phe	0.7597	0.046	1.344	0.426
2509	Val-Thr	0.9854	0.017	0.842	-0.249

3038	Val-Val	0.9784	0.013	1.729	0.790
7903	Yohimbine	0.6635	0.013	2.852	1.512
