# Supplementary material for: Systems-level exploitation of OxyR regulon unravels a potential antibacterial target in Pseudomonas aeruginosa
Source: Commun Biol. 2025 Sep 26;8:1370. doi: 10.1038/s42003-025-08770-w (PMC12475218; doi:10.1038/s42003-025-08770-w)
Supplement: Supplementary file 2 — Description of Additional Supplementary Materials [file 42003_2025_8770_MOESM2_ESM.pdf]

Description of Additional Supplementary Files

File name: Supplementary Data 1

Description: The source data underlying the graphs and charts in the figures
